# Wavefunction Optimization at the Complete Basis Set Limit with Multiwavelets and DMRG

**DOI:** 10.1021/acs.jpca.5c05836

**Published:** 2025-11-13

**Authors:** Martina Nibbi, Luca Frediani, Evgueni Dinvay, Christian B. Mendl

**Affiliations:** † School of CIT, Department of Computer Science, 9184 Technical University of Munich, Boltzmannstraße 3, 85748 Garching, Germany; ‡ Hylleraas Centre, Department of Chemistry, 8016 UiT University of Tromsø, The Arctic University of Norway, N-9037 Tromsø, Norway; § Institute for Advanced Study, 9184 Technical University of Munich, Lichtenbergstraße 2a, 85748 Garching, Germany

## Abstract

The density matrix renormalization group (DMRG) is a powerful numerical technique to solve strongly correlated quantum systems: it deals well with systems which are not dominated by a single configuration (unlike Coupled Cluster) and it converges rapidly to the Full Configuration Interaction (FCI) limit (unlike truncated Configuration Interaction (CI) expansions). In this work, we develop an algorithm integrating DMRG within the multiwavelet-based multiresolution analysis (MRA). Unlike fixed basis sets, multiwavelets offer an adaptive and hierarchical representation of functions, approaching the complete basis set limit to a specified precision. As a result, this combined technique leverages the multireference capability of DMRG and the complete basis set limit of MRA and multiwavelets. More specifically, we adopt a pre-existing Lagrangian optimization algorithm for orbitals represented in the MRA domain and improve its computational efficiency by replacing the original CI calculations with DMRG. Additionally, we substitute the reduced density matrices computation with the direct extraction of energy gradients from the DMRG tensors. We apply our method to small systems such H_2_, He, HeH_2_, BeH_2_ and N_2_. The results demonstrate that our approach reduces the final energy while keeping the number of orbitals low compared to FCI calculations on an atomic orbital basis set.

## Graphical abstract

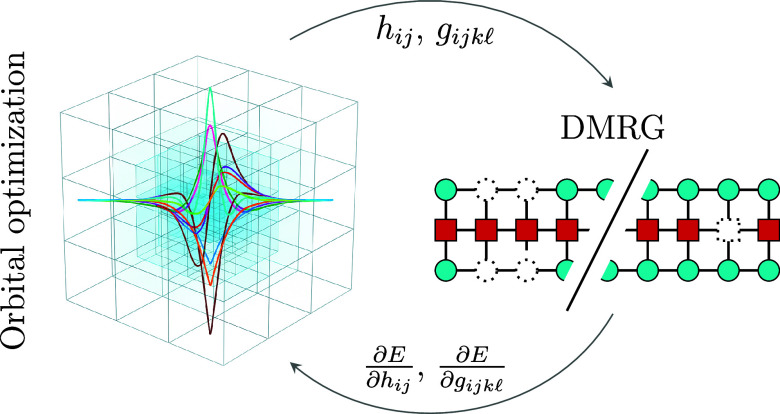

## 1 Introduction

The density matrix renormalization group (DMRG) is widely recognized as one of the most powerful numerical techniques for solving strongly correlated quantum systems.^1–3^ This success stems from its favorable computational scaling, polynomial in the number of orbitals for a fixed virtual bond dimension, combined with the ability to efficiently capture strong correlation and converge rapidly toward the Full Configuration Interaction (FCI) limit. As a result, DMRG offers much broader applicability than traditional Configuration Interaction or Coupled Cluster (CC) methods, which either converge slowly to FCI or are best suited to describe single-determinant dominated systems. Despite these advantages, the accuracy and efficiency of DMRG calculations strongly depend on the choice of the underlying orbital basis. Conventional basis sets, such as Gaussian Type Orbitals (GTOs) and plane waves (PWs), are often limited: the former converges slowly to the complete basis set (CBS) limit and is inherently nonorthonormal, whereas the latter has trouble describing the cusp region of the nuclei. Larger systems can also require an elevated number of orbitals to reach sufficient precision, which impacts the computational effort of DMRG.

In this context, multiwavelets (MWs) and multiresolution analysis (MRA) provide an appealing alternative. Wavelets and multiwavelets were first constructed in the 80’ of last century,^4^ with immediate application to signal processing, because they are localized both in real and in Fourier space.^5^ Unlike fixed basis sets, MWs offer an adaptive and hierarchical representation of functions and allow to reach the CBS limit up to any fixed, predefined precision.

Their ability to represent both localized and extended features of molecular wavefunctions makes them particularly well-suited for high-accuracy electronic structure calculations. This was first recognized at the end of the last century by Arias.^6^ A practical realization of electronic structure calculations using wavelets and multiwavelets was achieved a few years later. The BigDFT code features a wavelet representation of functions and operators, combined with a pseudopotential representation of the core electrons.^7^ It is capable of density functional theory (DFT) calculations of both isolated and periodic systems. The M-A-D-N-E-S-S code^8^ was the first to demonstrate the feasibility of all-electron calculations using MWs. To achieve an all-electron description, several technical advances are required, which operate in synergy to overcome the obstacles posed by a three-dimensional representation of functions:

1. self-consistent field (SCF) equations for orbital optimization are presented in an integral formulation, enabling the use of Green’s function technology;^9^
2. adaptive grids, which limit the memory footprint, are employed to represent functions;^10^
3. the Non-Standard (NS) form of the operator is required^11^ to preserve adaptivity when operators are applied;
4. operators are represented as a sum of separable terms (generally Gaussians) up to the requested precision,^12^ to limit the curse of dimensionality;
5. localized orbitals are employed,^13^ to achieve near-linear scaling with the system size.

All these advances have made it possible to use MWs for all-electron calculations at the SCF level.^8,13,14^ Nevertheless, introducing correlation has for a long time proven to be a formidable task: on the one hand the curse of dimensionality makes it much harder to represent functions in six dimensions, requiring compression techniques such as tensor-train decomposition for the representation of the two-body terms,^15^ or explicit R12 methods to describe the electron–electron cusp effectively.^16^ On the other hand, correlated methods are often based on exploiting the (finite) virtual space to go beyond the single-determinant representation. MWs do not make use of a finite virtual space and one has to devise strategies to obtain such corrections directly, which is possible only for a handful of cases such as MP2 ^17^ or CC2 ^18,19^ and is not of general applicability, because that would require a (finite) set of virtual orbitals.

In this respect, the recent work from Valeev et al.^20^ provides a general method to deal with correlated wavefunctions in a MW framework. The correlation problem is handled in a traditional way, namely a CI-type optimization, whereas the three-dimensional orbitals are represented with MWs. The approach relies on employing natural orbitals (NOs) which is a crucial step to ensure that orbital energies in the Helmholtz kernel will stay negative.

Integrating DMRG into the original optimization algorithm from Valeev et al.^20^ is a natural next step to improve its computational efficiency. Specifically, DMRG replaces the original CI calculations, and we introduce an alternative approach to the reduced density matrices (RDMs) evaluation by extracting energy gradients directly from the DMRG tensor network. This work also represents the first application of DMRG within the MRA and MWs framework.

In this paper, we begin by introducing the MWs framework as a basis for our method. Building on this, we derive the self-consistent equation for orbital optimization by combining MRA with DMRG. While the mathematical derivation follows the same structure as in Valeev et al.,^20^ we present it here for clarity. This also allows us to highlight certain implementation details that were overlooked in the original work but are crucial for computational efficiency. Numerical benchmarks on small systems show that this approach consistently yields lower energies than standalone DMRG or FCI calculations on atomic orbital basis sets, owing to the systematic completeness of the MWs basis.

## 2 Methods

### 2.1 Multiresolution Analysis and Multiwavelets

Alpert’s multiwavelets^21^ are a simple realization of MW functions which start from a set of *k* + 1 polynomials φ_*j*_(*x*) up to order *k* in the interval (0, 1) and zero otherwise. They constitute a vector space *V*_0_. Starting from *V*_0_ one can then obtain a ladder of nested spaces *V*_*n*_, with *n* = 1, 2, 3··· by dilation and translation of the original functions:

$$\varphi_{j,l}^{n}\left(x\right)=2^{n/2}\varphi_{j}\left(2^{n}x-l\right)$$ 1

It can be shown that the sequence of nested spaces *V*_0_ ⊂ *V*_1_ ⊂ *V*_2_ ⊂ *V*_3_ ⊂··· is dense in *L* ^2^. Wavelet spaces are then constructed by taking the orthogonal complement between two successive scaling spaces, such that *V*_*n*_ ⊕ *W*_*n*_ = *V*_*n*+1_.

Moreover, by using MWs one can obtain sparse representations of functions^22^ and certain convolution-type operators,^23^ such as the Helmholtz operator:

$${\hat{G}}_{-\lambda }f(\mathrm{r⃗})=-2\int \frac{e^{-\sqrt{2\lambda }|\mathrm{r⃗}-{\mathrm{r⃗}}^{\prime }|}}{4\pi |\mathrm{r⃗}-{\mathrm{r⃗}}^{\prime }|}f({\mathrm{r⃗}}^{\prime })\;d{\mathrm{r⃗}}^{\prime },,\lambda >0$$ 2

This leads to fast and efficient algorithms by maintaining control over the representation error,^10^ which is the distinctive feature of MW algorithms.

In Figure 1 we report a graphical representation of the concepts mentioned so far.^24^ It is beyond the scope of this paper to give a comprehensive exposition of MW methods, which have been presented in detail in the literature.^25^

**Figure 1:**
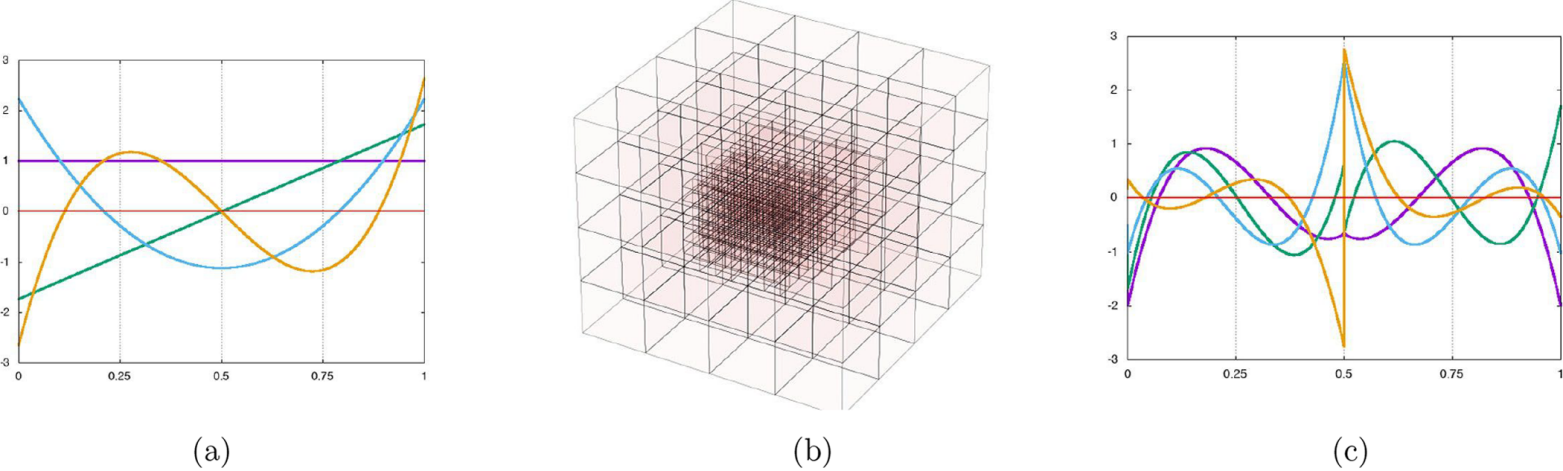
(a) Scaling functions in *V*_0_ for *k* = 3. (b) Multireference grid in 3D space. (c) MWs in *W*_0_ for *k* = 3. Reproduced with permission from.^24^

### 2.2 Combining MRA with DMRG

In this section, we show how to combine MRA with DMRG for orbital optimization. Our method takes inspiration from the work of Valeev et al.,^20^ which consists of a multireference technique using MRA and MWs.

Given a finite set of real orbitals {ϕ_*m*_}, we first build the second-quantized chemical Hamiltonian:

$$\hat{H}=\sum_{i,j}\sum_{\sigma \in \{↑,↓\}}h_{ij}{\hat{a}}_{i,\sigma }^{†}{\hat{a}}_{j,\sigma }+\frac{1}{2}\sum_{i,j,k,l}\sum_{\sigma ,\tau }g_{ijkl}{\hat{a}}_{i,\sigma }^{†}{\hat{a}}_{j,\tau }^{†}{\hat{a}}_{l,\tau }{\hat{a}}_{k,\sigma }$$ 3

=ĥ+ĝ 4

where *ĥ* and *ĝ* are the one- and two-body operators and with one- and two-body integrals respectively defined as

$$h_{ij}=\int \phi_{i}({\mathrm{r⃗}}^{\prime })\left[-\frac{\nabla^{2}}{2}+\sum_{\alpha }\frac{Z_{\alpha }}{\left|{\mathrm{r⃗}}^{\prime }-{\mathrm{R⃗}}_{\alpha }\right|}\right]\phi_{j}({\mathrm{r⃗}}^{\prime })\;d{\mathrm{r⃗}}^{\prime }$$ 5

$$g_{ijkl}=\int \phi_{i}({\mathrm{r⃗}}^{\prime })\phi_{j}({\mathrm{r⃗}}^{\prime \prime })\frac{1}{\left|{\mathrm{r⃗}}^{\prime }-{\mathrm{r⃗}}^{\prime \prime }\right|}\phi_{l}({\mathrm{r⃗}}^{\prime \prime })\phi_{k}({\mathrm{r⃗}}^{\prime })\;d{\mathrm{r⃗}}^{\prime }\;d{\mathrm{r⃗}}^{\prime \prime }$$ 6

We aim to find the ground-state |Φ_GS_⟩ and the corresponding energy *E* = ⟨Φ_GS_|*Ĥ*|Φ_GS_⟩ through a DMRG calculation (see Appendix A). However, this ground-state energy estimate heavily relies on the choice of orbitals {ϕ_*m*_} defining *Ĥ*. As a consequence, in order to get a good energy estimate, we also have to optimize the underlying orbitals.

Techniques involving atomic orbitals rely on a fixed basis, while the present method optimizes the orbitals at the CBS limit. In particular, we want to optimize the orbitals while preserving their orthogonality. This translates to a constraint optimization with Lagrangian:

$$L=E-\underset{i,j}{\sum }\varepsilon_{ij}\left(s_{ij}-\delta_{ij}\right)$$ 7

where *s* is the orbitals’ overlap matrix and ε is the Lagrange multiplier matrix.

Following Valeev et al.,^20^ we differentiate L with respect to variations of the orbitals ϕ_*m*_, we get:

$$\left|\frac{\partial L}{\partial \phi_{m}}\right\rangle =0$$ 8

$$\frac{\partial E}{\partial \phi_{m}}-\underset{ij}{\sum }\varepsilon_{ij}\frac{\partial s_{ij}}{\partial \phi_{m}}=0$$ 9

The first term in eq 9 can be expanded by using the chain rule:

$$\frac{\partial E}{\partial \phi_{m}}=\underset{ij}{\sum }\frac{\partial h_{ij}}{\partial \phi_{m}}\frac{\partial E}{\partial h_{ij}}+\underset{ijkl}{\sum }\frac{\partial g_{ijkl}}{\partial \phi_{m}}\frac{\partial E}{\partial g_{ijkl}}$$ 10

where *h*_*ij*_ and *g*_*ijkl*_ are defined in eqs 5 and 6, respectively.

This notation is particularly convenient because the terms ∂*E*/∂*h*_*ij*_ and $\partial E/\partial g_{ijkl}$ can be easily derived by “cutting holes” in the matrix product operator (MPO) used for the DMRG calculation, as shown in Appendix A. It is worth remarking that instead of the energy gradients, Valeev et al.^20^ evaluated the one- and two-body RDMs. While calculated in a different way, it can easily be proven that these quantities are equivalent:

$$\frac{\partial E}{\partial h_{ij}}=\langle \Phi_{\mathrm{GS}}|{Ê}_{ij}|\Phi_{\mathrm{GS}}\rangle =\gamma_{ij}$$ 11a

$$\frac{\partial E}{\partial g_{ijkl}}=\langle \Phi_{\mathrm{GS}}|{ê}_{ijkl}|\Phi_{\mathrm{GS}}\rangle =\gamma_{ijkl}$$ 11b

where the spin-free 1-electron and 2-electron excitation operators are defined as *Ê*_*ij*_ = ∑_σ_ *a*_*i*,σ_^†^ *a*_*j*,σ_ and *ê*_*ijkl*_ = *Ê*_*ik*_ *Ê*_*jl*_ – δ_*jk*_ *Ê*_*il*_ = ∑_σ,τ_ *a*_*i*,σ_^†^ *a*_*j*,τ_^†^ *a*_*l*,τ_ *a*_*k*,σ_, following the conventions used by Helgaker, Jørgensen and Olsen.^26^

As a result, our mathematical derivation is also equivalent. However, we chose to present the same proof again as this allows us to focus more on the implementation details, which were sometimes missing from the original work and are fundamental when taking efficiency into account.

The first step consists of the analytic calculation of ∂*h*_*ij*_/∂ϕ_*m*_ and $\partial g_{ijkl}/\partial \phi_{m}$ . Specifically, for the one-body term we get

$$\frac{\partial h_{ij}}{\partial \phi_{m}}=\delta_{im}ĥ|\phi_{j}\rangle +\delta_{jm}ĥ|\phi_{i}\rangle$$ 12

The first term of eq 10 then looks like

$$\sum_{ij}\frac{\partial h_{ij}}{\partial \phi_{m}}\frac{\partial E}{\partial h_{ij}}=\sum_{j}\left[\frac{\partial E}{\partial h_{mj}}+\frac{\partial E}{\partial h_{jm}}\right]ĥ|\phi_{j}\rangle =2\sum_{j}\partial E_{mj}^{\left(1\right)}ĥ|\phi_{j}\rangle$$ 13

where ∂*E* ^(1)^ is the symmetrized version of the one-body gradient:

$$\partial E^{\left(1\right)}=\frac{1}{2}\left[\frac{\partial E}{\partial h}+{\left(\frac{\partial E}{\partial h}\right)}^{T}\right]$$ 14

Similarly, for what concerns the two-body term, we derive

$$\frac{\partial g_{ijkl}}{\partial \phi_{m}}=\delta_{im}{\hat{g}}_{jl}|\phi_{k}\rangle +\delta_{jm}{\hat{g}}_{ik}|\phi_{l}\rangle +\delta_{km}{\hat{g}}_{jl}|\phi_{i}\rangle +\delta_{lm}{\hat{g}}_{ik}|\phi_{j}\rangle$$ 15

where the operator *ĝ*_*jl*_ is defined as

$${\hat{g}}_{jl}=\int \phi_{j}({\mathrm{r⃗}}^{\prime })\frac{1}{\left|\mathrm{r⃗}-{\mathrm{r⃗}}^{\prime }\right|}\phi_{l}({\mathrm{r⃗}}^{\prime })\;d{\mathrm{r⃗}}^{\prime }$$ 16

The second term of eq 10 can then be written as

$$\sum_{ijkl}\frac{\partial g_{ijkl}}{\partial \phi_{m}}\frac{\partial E}{\partial g_{ijkl}}=\sum_{jkl}\left[\frac{\partial E}{\partial g_{mjkl}}+\frac{\partial E}{\partial g_{jmlk}}+\frac{\partial E}{\partial g_{kjml}}+\frac{\partial E}{\partial g_{jklm}}\right]{ĝ}_{jl}|\phi_{k}\rangle =4\sum_{jkl}\partial E_{mjkl}^{\left(2\right)}{ĝ}_{jl}|\phi_{k}\rangle$$ 17

where ∂*E* ^(2)^ is the symmetrized version of the two-body gradient:

$$\partial E^{\left(2\right)}=\frac{1}{4}\left[\frac{\partial E}{\partial g}+{\left(\frac{\partial E}{\partial g}\right)}^{T_{1,0,3,2}}+{\left(\frac{\partial E}{\partial g}\right)}^{T_{2,1,0,3}}+{\left(\frac{\partial E}{\partial g}\right)}^{T_{1,2,3,0}}\right]$$ 18

in which the transposition $T_{i,j,k,l}$ switches the indices 0 → *i*, 1 → *j*, 2 → *k* and 3→l .

Finally, we apply the same derivation to the multipliers’ term in eq 9:

$$\sum_{ij}\varepsilon_{ij}\frac{\partial s_{ij}}{\partial \phi_{m}}=\sum_{ij}\varepsilon_{ij}\frac{\partial }{\partial \phi_{m}}\int \phi_{i}({\mathrm{r⃗}}^{\prime })\phi_{j}({\mathrm{r⃗}}^{\prime })\;d{\mathrm{r⃗}}^{\prime }=\sum_{ij}(\delta_{im}|\phi_{j}\rangle +\delta_{mj}|\phi_{i}\rangle )\varepsilon_{ij}=\sum_{j}(\varepsilon_{jm}+\varepsilon_{jm})|\phi_{j}\rangle =2\sum_{j}{\mathrm{\varepsilon ̅}}_{mj}|\phi_{j}\rangle$$ 19

with the symmetrized multipliers matrix defined as

$$\mathrm{\varepsilon ̅}=\frac{1}{2}[\varepsilon +\varepsilon^{T}]$$ 20

As a result, we get the following expression for the vanishing gradients of the Lagrangian w.r.t. the orbitals:

$$\sum_{j}(\partial E_{mj}^{\left(1\right)}ĥ-{\overset{®}{\varepsilon }}_{mj})|\phi_{j}\rangle +2\sum_{jkl}\partial E_{mjkl}^{\left(2\right)}{ĝ}_{jl}|\phi_{k}\rangle =0$$ 21

We can derive the Lagrangian multipliers by projecting eq 21 on the orthonormal orbitals:

$$\left\langle \phi_{n}\right|\frac{\partial L}{\partial \phi_{m}}\rangle =0$$ 22

from which we get

$${\mathrm{\varepsilon ̅}}_{mn}=\sum_{j}\partial E_{mj}^{\left(1\right)}h_{nj}+2\sum_{jkl}\partial E_{mjkl}^{\left(2\right)}g_{njkl}$$ 23

To simplify the equations, Valeev et al.^20^ used NOs, which diagonalize the one-body RDM. We achieve the same outcome, by diagonalizing the one-body energy gradient. Given then the basis-change matrix *U* such that

$$\partial E^{\left(1\right)}=U^{†}\Lambda U$$ 24

where Λ is diagonal, we can derive eq 21 in the new basis for every *m*:

$$\Lambda_{m}ĥ|\phi_{m}^{\prime }\rangle +2\sum_{ijkl}U_{mi}\partial E_{ijkl}^{\left(2\right)}{ĝ}_{jl}|\phi_{k}\rangle -\sum_{j}\varepsilon_{mj}^{\prime }|\phi_{j}^{\prime }\rangle =0$$ 25

where:

$$\left|\phi_{i}^{\prime }\right\rangle =\underset{j}{\sum }U_{ij}\left|\phi_{j}\right\rangle$$ 26

and:

$$\varepsilon^{\prime }=U\mathrm{\varepsilon ̅}U^{†}$$ 27

The two-body term is not rotated because the summation is carried out over the whole set. We notice in this respect that this flexibility could be exploited to compute the two-body term on a localized basis to achieve a reduced scaling of this time-consuming step.

Finally, we obtain the self-consistent equation:

$$-\left(\hat{d}-\frac{\varepsilon_{mm}^{\prime }}{\Lambda_{m}}\right)|\phi_{m}^{\prime }\rangle =\hat{v}|\phi_{m}^{\prime }\rangle +\frac{1}{\Lambda_{m}}\left(2\sum_{ijkl}U_{mi}\partial E_{ijkl}^{\left(2\right)}{\hat{g}}_{jl}|\phi_{k}\rangle -\sum_{j\neq m}\varepsilon_{mj}^{\prime }|\phi_{j}^{\prime }\rangle \right)$$ 28

where the one-body operator *ĥ* = *d̂* + *v̂* has been split into its kinetic and potential contributions.

The orbital update equation is then derived by inverting eq 28:

$$|\phi_{m}^{\prime }\rangle =-{Ĝ}_{\varepsilon_{mm}^{\prime }/\Lambda_{m}}\left[\hat{v}|\phi_{m}^{\prime }\rangle +\frac{1}{\Lambda_{m}}\left(2\sum_{ijkl}U_{mi}\partial E_{ijkl}^{\left(2\right)}{ĝ}_{jl}|\phi_{k}\rangle -\sum_{j\neq m}\varepsilon_{mj}^{\prime }|\phi_{j}^{\prime }\rangle \right)\right]$$ 29

where the Helmholtz operator *Ĝ*_–λ_ was defined in eq 2.

It is worth remarking that the inversion of eq 28 is practical only if the coefficients ε_*mm*_ *′*/Λ_*m*_ are negative. By rewriting eq 23 in the new orbitals’ basis, we get:

$$\frac{\varepsilon_{mm}^{\prime }}{\Lambda_{m}}=h_{mm}^{\prime }+2\underset{jkl}{\sum }\frac{U_{mi}U_{mn}\partial E_{ijkl}^{\left(2\right)}g_{njkl}}{\Lambda_{m}}$$ 30

where *h*_*mm*_ *′* is the one-body integral in the new orbitals’ basis and *U* is the base change matrix from eq 24. Valeev et al.^20^ came to the same definition of the Helmholtz kernel’s coefficients and they argued that, at least empirically, they always tend to be negative since *h*_*mm*_ *′* is always negative and the second term in eq 30 is small in absolute value. While this is not a mathematical proof, we have also experienced it to hold for most of the numerical tests we have performed.

In conclusion, we were able to derive the same self-consistent working equation as Valeev et al.^20^ However, while they evaluated the RDMs through a Heat-Bath CI calculation,^27^ we can obtain the same quantities at a much lower computational effort through a DMRG calculation, which also provides a precise energy estimate through the entire optimization process. The orbitals’ optimization algorithm combining MRA and DMRG is then summarized in Algorithm 1 and in the flowchart in Figure 2, where we have also highlighted which steps are based on the MRA representation and which can be considered orbital-agnostic. This constitutes the first algorithm ever leveraging DMRG in a MRA and MWs framework. Note also that, unlike the original work, we perform a Löwdin orthonormalization of the orbitals^28^ as a final step of each iteration in order to smoothen the convergence.
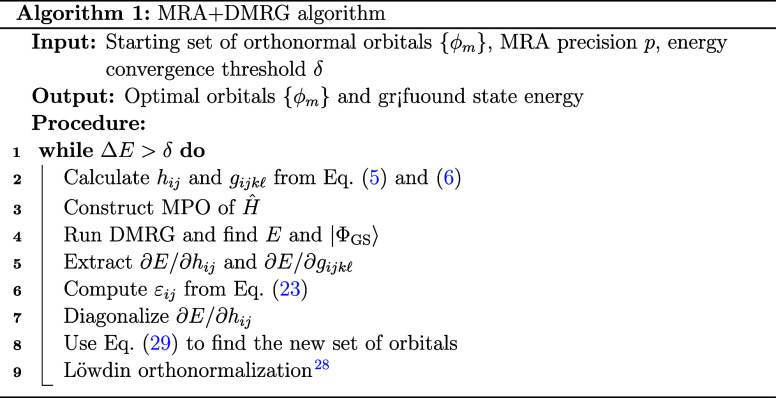


**Figure 2:**
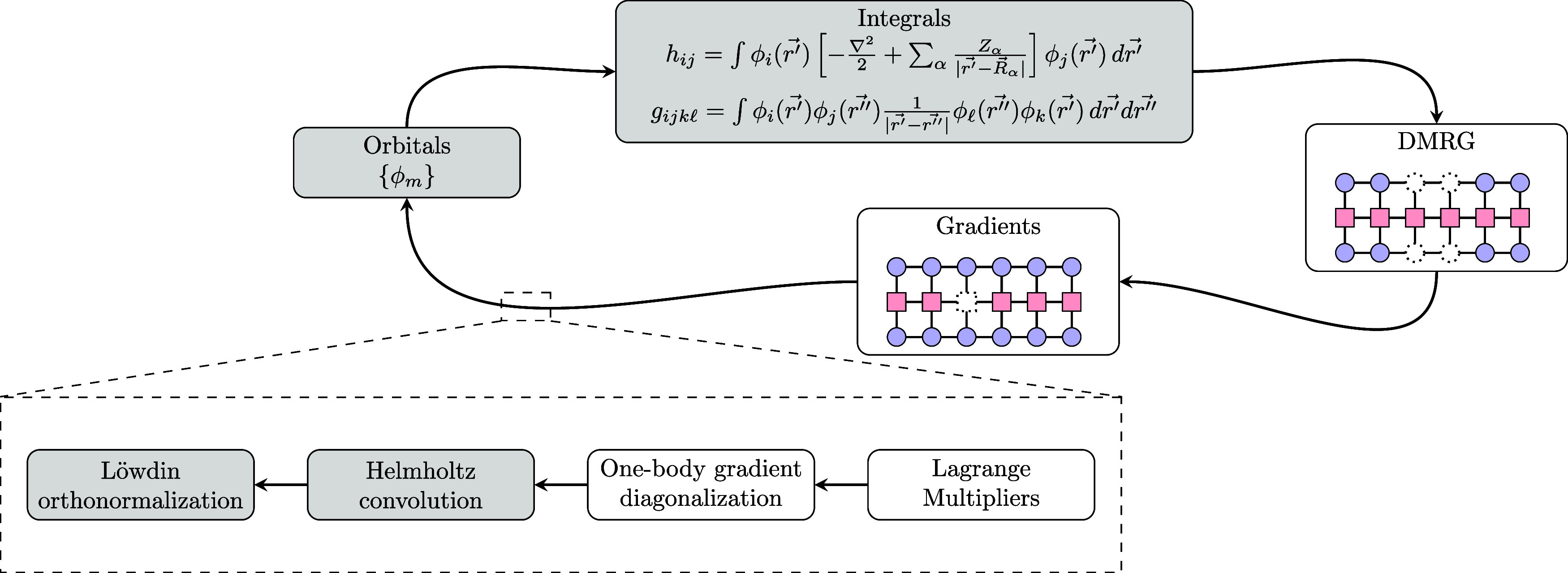
MRA+DMRG flowchart. The steps colored in gray belong to the “MRA domain”. The white blocks are orbital-agnostic.

## 3 Results and Discussion

We have tested our algorithm on a few examples, where correlation effects are known to play an important role^29,30^ despite their small size, and we report a comparison with the Hartree–Fock method plus the pure application of DMRG and FCI acting on an atomic orbital basis set. All MRA calculations have precision *p* = 10^–5^ and polynomial order *k* = 9. Moreover, we have fixed the energy convergence threshold δ = 10^–5^ Ha. All the experiments were conducted on a workstation computer equipped with an AMD EPYC 7402 CPU 2.80 GHz, 24 cores, and 256 GB RAM.

For MRA and MWs representation, we have relied on the Python software VAMPyR,^31^ which is a Python interface to the underlying C++ mathematical library MRCPP.^32^ At the same time, DMRG and gradient extraction are implemented in the chemtensor^33^ library and are described in Appendix A. Neither library is currently optimized for a high-performance computing (HPC) framework, which prevented us from considering larger systems. Therefore, we only provide results for up to 15 orbitals, while we leave the task of optimizing the code for future work. The Hartree–Fock (HF) and FCI energies have been calculated with Dalton^34^ with atomic basis sets up to 28 orbitals.

The results can be found in Figure 3 and among all the molecules we have tested, we can provide the following remarks:

- MRA eliminates the need for a predefined atomic basis. This allows for calculations with any specified number of orbitals, for which there might be no corresponding atomic orbital basis set.
- The simple DMRG calculation matches with FCI, often with precision beyond the eighth decimal digit. For such small systems, this was entirely expected and serves as a benchmark for chemtensor.
- In every scenario, and while maintaining a fixed number of orbitals, our algorithm shows significant improvements in the final energy. This effect is especially evident in the N_2_ example with 10 orbitals, where the MRA+DMRG algorithm yields a 442 mHa energy reduction compared to FCI using a fixed atomic orbital basis set.
- Sometimes, the MRA+DMRG algorithm outperforms the standard FCI on an atomic orbital basis set even when increasing the basis size of the latter. Considering again the N_2_ example, the 10 and 12 orbitals’ final results are still more accurate than FCI with 6–31G basis (and 18 orbitals). Further evidence of this effect can be seen in the BeH_2_ calculations, where the 15 orbitals’ MRA+DMRG final energy constitutes a 44 mHa improvement compared to the FCI calculation with the cc-pVDZ basis (and 24 orbitals).
- As expected, such improvements tend to saturate when increasing the number of orbitals. This can be seen, for example, in the H_2_ simulation, where the energies of MRA+DMRG with 10 and 12 orbitals and FCI with 28 orbitals differ by less than 1 mHa.

These results reflect the fact that our method works at the CBS limit, up to any predefined precision *p*, while the standard implementation of HF, DMRG and FCI relies on a finite atomic orbital basis set. Notably, the computational scaling of DMRG depends only on the number of orbitals, not on the basis size. As a result, our method holds the promise of improving DMRG by increasing the basis size up to the CBS limit while preserving the number of orbitals and, thus, limiting its computational complexity.

**Figure 3:**
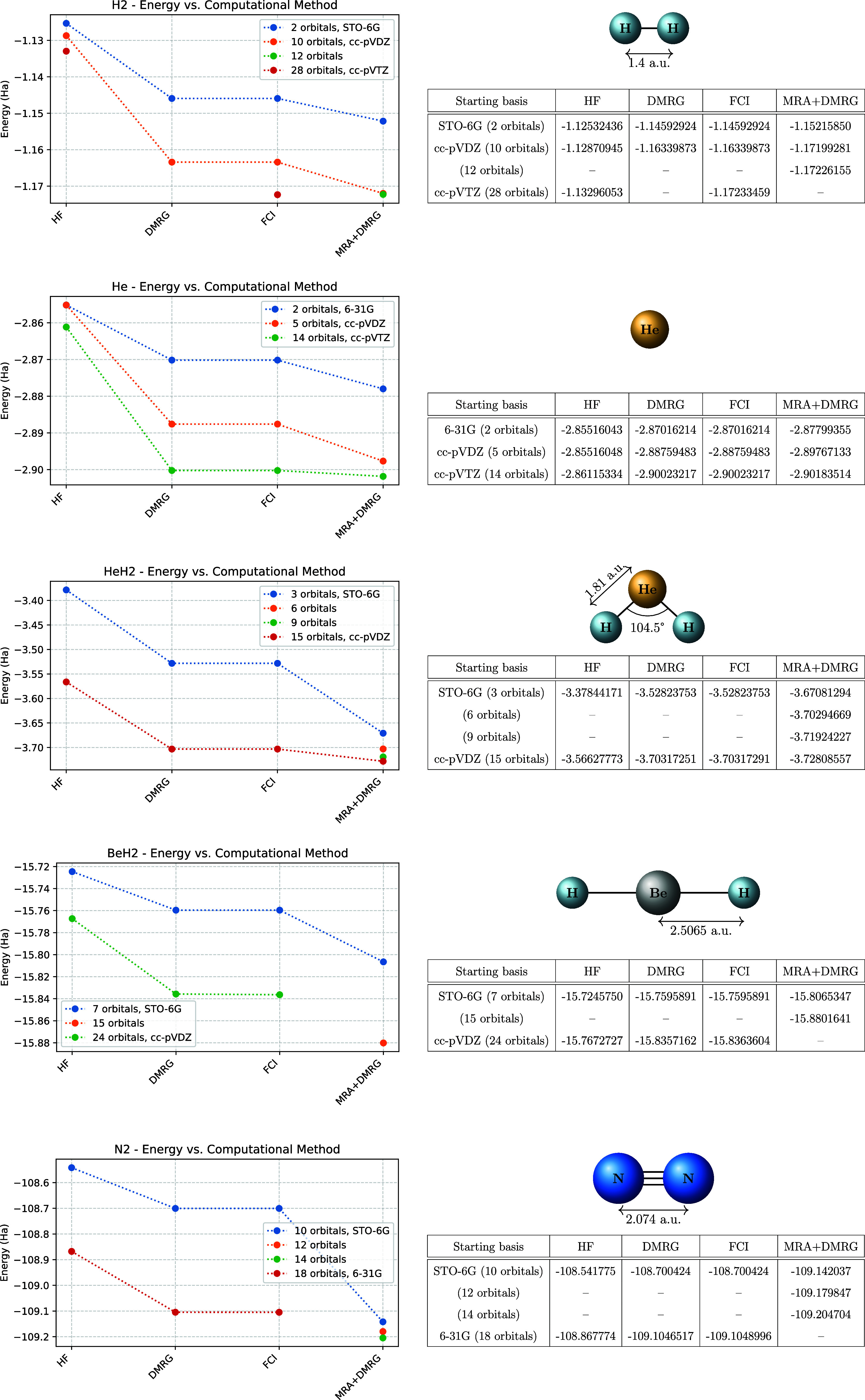
Final energies obtained from HF, DMRG, FCI, and our MRA+DMRG method. When using MRA, a precision of 10^–5^ was used with polynomial order *k* = 9. MRA+DMRG works in principle with any number of orbitals, but the current pilot implementation is limited to 15 orbitals. For AO calculations, the number of orbitals is dictated by the choice of basis. For these reasons, the tables present empty entries.

In Appendix B we have reported some of the convergence plots for the molecules tested in this section. For such systems, we observed smooth convergence and the energy parameters defining the Helmholtz kernel in eq 30 are found to be negative. This aligns with the empirical observation that ε_*mm*_ *′*/Λ_*m*_ tends to be negative, a necessary condition for the algorithm to function correctly.

It is worth noting that the MRA+DMRG algorithm qualifies as an ab initio method. While it is theoretically possible to combine it with methods such as Hartree–Fock or DFT, we did not need to perform any preliminary approximate calculation in the considered systems. Moreover, our method seems to have only a weak dependence on the choice of initial orbitals. We have used different Gaussian basis sets to build the starting wavefunctions, as well as Slater-type orbitals, but no practical difference has been observed in the final energies and the convergence paths. While this remains to be tested on larger systems, it appears to be a promising feature of such an ab initio method.

Additionally, in Figure 4, we have plotted the Hydrogen and Nitrogen dissociation energy paths as a further benchmark of our method: they show the correct asymptotic behavior that one expects from the proper multiconfigurational treatment.

**Figure 4:**
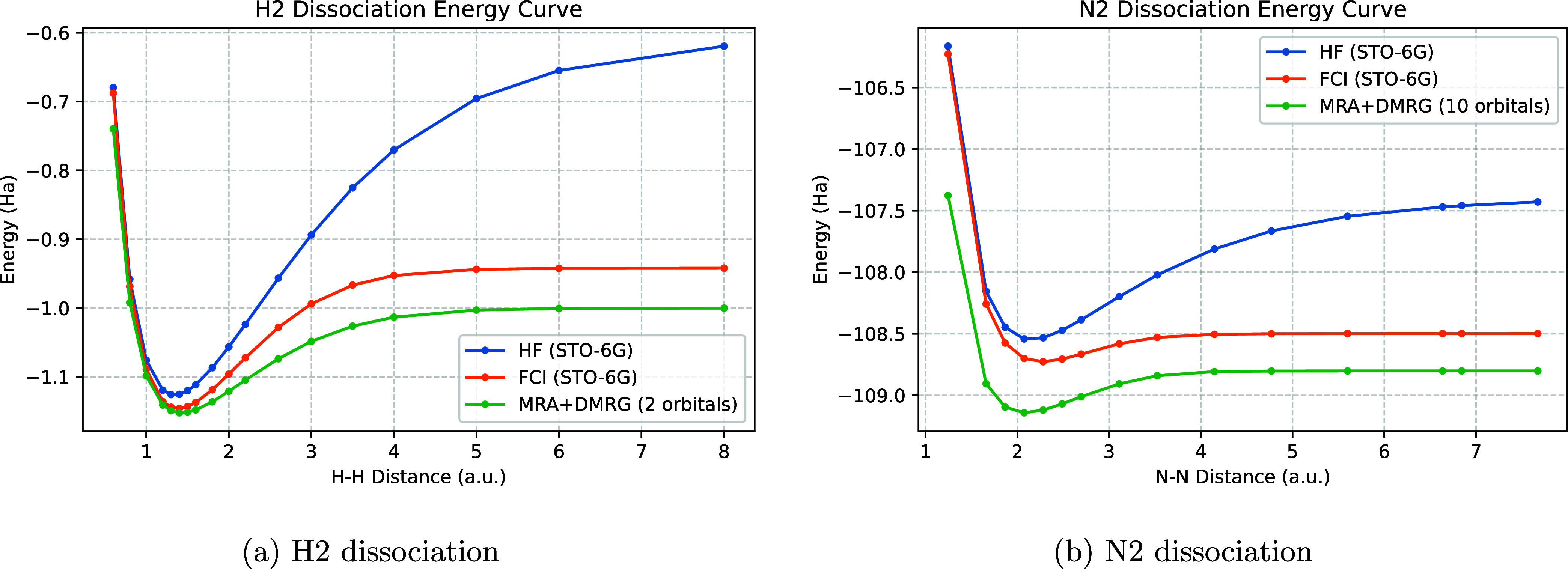
Dissociation paths of H2 (a) and of N2 (b). The ground-state energies are obtained via HF and FCI with a minimal basis set and the MRA+DMRG algorithm with the same number of orbitals, respectively.

## 4 Conclusions

This work represents a first attempt to apply DMRG within the MRA and MWs framework. Specifically, we have developed an ab initio and self-consistent orbital optimization algorithm inspired by the work of Valeev et al.^20^ While following a similar workflow, our method replaces CI energy estimates with DMRG and extracts energy gradients directly from the DMRG tensor network, avoiding the need for RDM evaluations, as described in Appendix A.

We have tested our approach on small molecules with up to 15 orbitals, with results presented in Section 3. Our method consistently achieves lower energies for a fixed number of orbitals than standalone FCI or DMRG, benefiting from the larger basis set. This demonstrates that the combination of MRA and DMRG successfully captures the multireference nature of the system while approaching the infinite basis set limit, with MRA precision *p* and the number of orbitals as the primary limitations.

Despite its potential, our approach is currently limited to small systems with a restricted number of orbitals due to computational limitations. For the MRA calculations, we relied on the Python package VAMPyR,^31^ which, while user-friendly, is not optimized for an HPC framework. Future work will focus on migrating to the more efficient MRChem^13^ and optimizing chemtensor^33^ for HPC integration.

As mentioned in Appendix B, all cases considered show a smooth convergence up to energy precision δ = 10^–5^ Ha. In the future, we aim to consider larger systems, which may exhibit slower or more difficult convergence. In this regard, the algorithm could be improved through a Newton optimization method,^35^ which would further reduce its dependence on the initial guess.

Another unexplored aspect is orbital localization. Localized orbitals yield a more compact MRA representation, reducing the computational cost. DMRG calculations also benefit from localization in strongly correlated systems.^36^ For these reasons, incorporating a localization step into the algorithm could enhance its overall efficiency. At present, however, it is unclear to what extent localization can be exploited. The current algorithm is designed around the diagonalization of the one-body RDM, which restricts the orbital basis to the NOs.^37^ This constraint is absent in standard SCF calculations, where localization can be applied more freely. Natural orbitals are not necessarily localized, although adaptations to improve locality have been proposed.^38^ Moreover, recent work by one of the authors^39^ has shown that the explicit application of the Laplacian operator, which is currently avoided by diagonalizing the one-body RDM, can be performed efficiently. This would allow different orbital choices, thus enabling a more systematic exploitation of locality.

During this project, we became aware of the work by Langkabel et al.,^40^ who also built upon the Lagrangian optimization from Valeev et al.^20^ but replaced CI calculations with a variational quantum eigensolver (VQE).^41^ In future work, it would be interesting to compare these two techniques and explore their respective regimes of applicability. This also highlights the growing interest in MRA and MWs techniques within different branches of quantum chemistry.

Our work is an initial step in bridging two distinct approaches: multiresolution analysis and tensor network applications in chemistry. The presented results seem promising; however, further optimization is needed to assess the algorithm’s feasibility in larger and more complex systems.

## Figures and Tables

**Figure 5:**
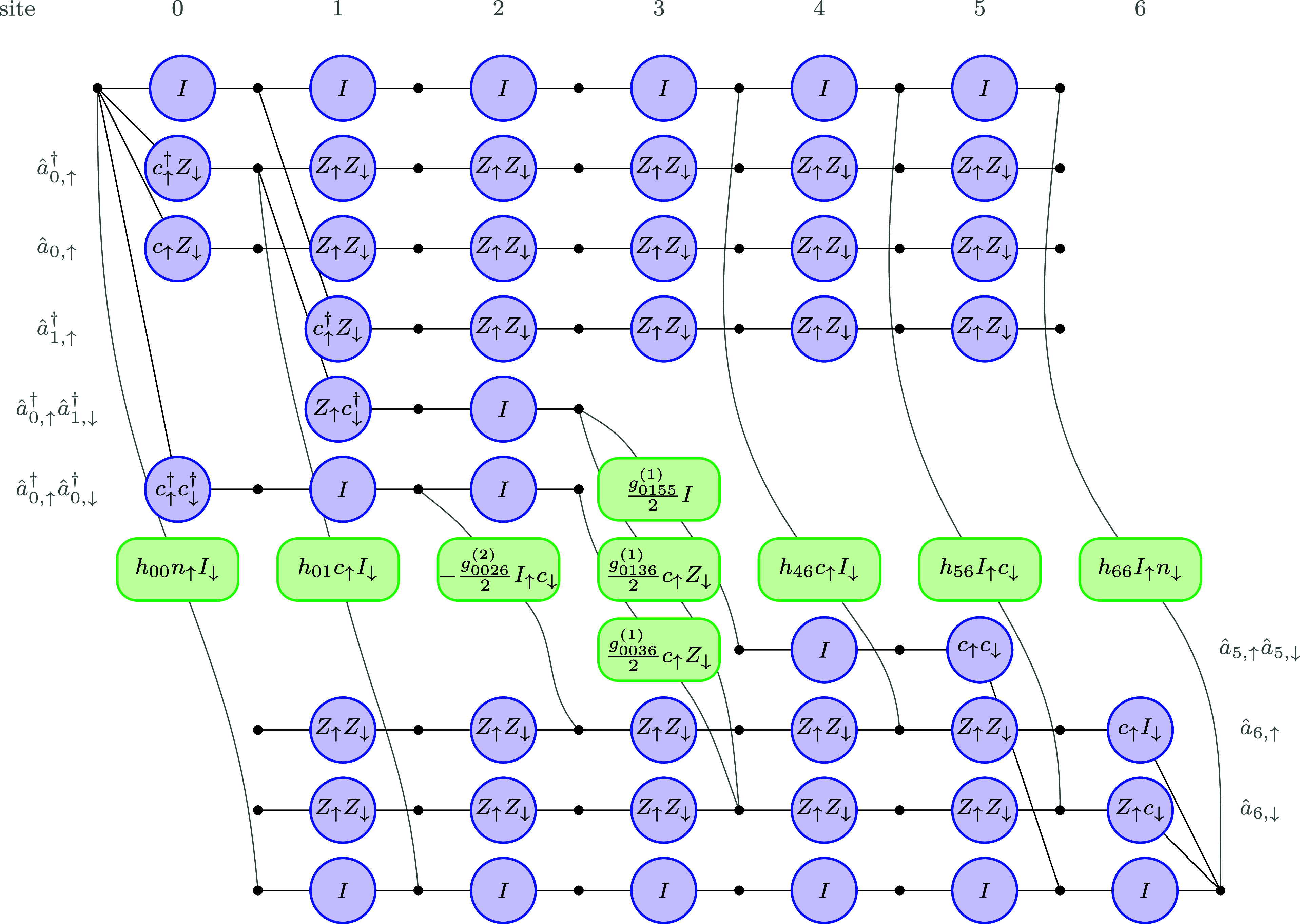
State diagram for constructing the MPO representation of a molecular Hamiltonian ([Disp-formula eq32]). *c*_σ_^†^ and *c*_σ_ for σ ∈{↑, ↓} are the local bosonic creation and annihilation operators, *Z*_σ_ is the Pauli-*Z* gate acting on spin sector σ ∈{↑, ↓}, and *I* is the identity operation. The “scaffolding” is highlighted in blue. The figure only shows a few operator strings and Hamiltonian terms for visual clarity.

**Figure 6:**
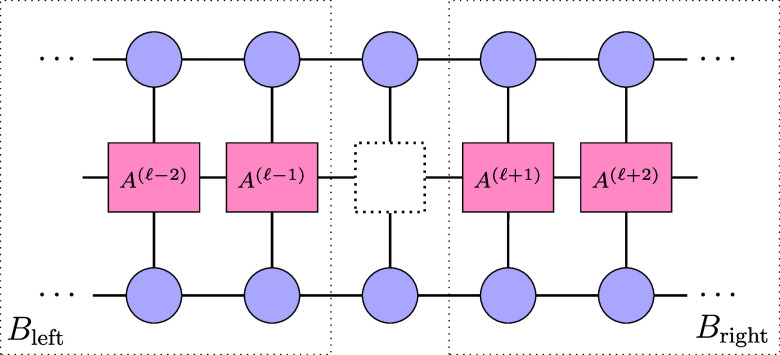
Extraction of $\left\langle \psi |\frac{\partial Ĥ}{\partial A_{J}^{\left(l\right)}}|\psi \right\rangle$ for ∀*J* for the energy gradients calculation. The environment blocks *B*_left_ and *B*_right_ can be precomputed for efficiency.

**Figure 7:**
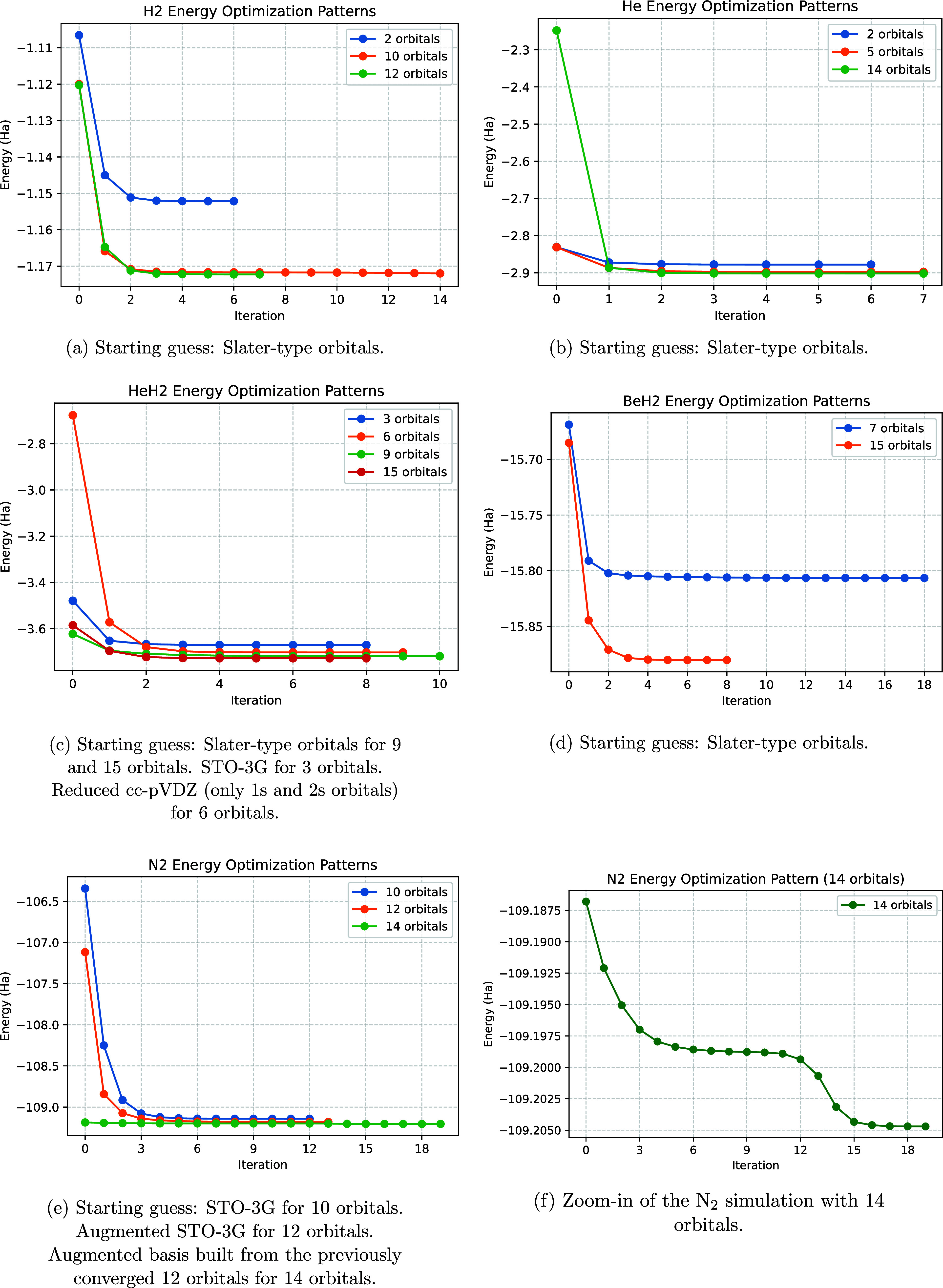
Convergence patterns of H_2_ (a), He (b), HeH_2_ (c), BeH_2_ (d), and N_2_ (e,f) with energy threshold δ = 10^–5^ Ha. The initial guesses are specified for each system.

## Appendix A: DMRG and Gradients

The DMRG algorithm^1,42^ is a tensor network method suitable for approximating the ground state of (strongly correlated) quantum systems in condensed matter physics and chemistry.^2,3,36,43–45^ DMRG uses a matrix product state (MPS) as a variational Ansatz for the wavefunction and can reach energies close to the exact (FCI) ground state. For a fixed virtual bond dimension, DMRG exhibits a computational cost that scales polynomially with the number of orbitals. While the required bond dimension remains modest in many practical scenarios, this is not guaranteed, and its growth in strongly correlated systems is still poorly understood and difficult to predict.

The molecular Hamiltonian in eq 3 exhibits abelian symmetries corresponding to global particle number and spin conservation. In general, such abelian symmetries endow the MPS and MPO tensors with a block sparsity structure, which can be advantageous to increase computational performance.^46^ In the current setting, we associate a particle number and spin tuple (*q*_*n*_, *q*_*s*_) with each index at every tensor leg. The tensor legs also have a predefined direction, either outgoing or incoming. The conservation laws then require that the sum of incoming quantum numbers must be equal to the sum of outgoing quantum numbers; otherwise, the respective tensor entry has to be zero. We use the convention that each local “site” *l* has dimension 4, corresponding to the possible occupation patterns of the two spin orbitals $\left\{\phi_{l,↑},\phi_{l,↓}\right\}$ , and enumerate these patterns as follows:The sparsity condition of the tensors can be illustrated for an individual MPS tensor:The inner blue numbers define the tensor leg order. The horizontal legs (0 and 2) are the virtual bonds, and the vertical leg (1) is the physical dimension. Each leg stores a separate list of quantum number tuples. For example, for the physical leg, these are

$${\left\{\left(q_{n,j}^{\left(1\right)},q_{s,j}^{\left(1\right)}\right)\right\}}_{j=0,...,3}=\left\{\left(0,0\right),\left(1,-\frac{1}{2}\right),\left(1,\frac{1}{2}\right),\left(2,0\right)\right\}$$

according to the above table, where the superscript indicates the tensor leg. The sparsity condition means that the entry *A*_*ijk*_ can be nonzero only if

$$q_{n,i}^{\left(0\right)}+q_{n,j}^{\left(1\right)}=q_{n,k}^{\left(2\right)}$$
31a

$$q_{s,i}^{\left(0\right)}+q_{s,j}^{\left(1\right)}=q_{s,k}^{\left(2\right)}$$
31b

i.e., if the quantum number “inflow” matches the “outflow”.

Constructing an MPO representation of the molecular Hamiltonian with optimal virtual bond dimension (scaling as $O\left(L^{2}\right)$ for *L* orbitals) is intricate in detail. A recipe reported in the literature is the so-called complementary operator approach.^44^ Here, we sketch an alternative (but equivalent) viewpoint and procedure, illustrated in Figure 5. The idea is to construct a “scaffolding” of creation and annihilation operator strings, then synthesize the Hamiltonian terms by connecting these strings with appropriate local operators. The graph-like data structure, denoted state diagram and described in,^47,48^ helps organize this construction and track terms. The black dots in Figure 5 are interpreted as vertices of a graph; each vertex becomes a virtual bond in the final MPO. The circles are the graph’s edges and contain local operators, which populate the final MPO tensors. We use the Jordan-Wigner transformation to account for the Fermionic anticommutation relations. In practice, this means inserting Pauli-*Z* gates between odd occurrences of the creation and annihilation operators.

More in detail, we can first combine the terms appearing in the interaction part of the Hamiltonian ([Disp-formula eq3]) as follows:

$$\hat{H}=\sum_{i,j}\sum_{\sigma \in \{↑,↓\}}h_{ij}{â}_{i,\sigma }^{†}{â}_{j,\sigma }+\frac{1}{2}\sum_{\begin{array}{c} (i,\sigma )<(j,\tau ),\\ \left(k,\sigma^{\prime })<(l,\tau^{\prime }\right) \end{array}}(g_{ijkl}^{\left(1\right)}\delta_{\sigma ,\sigma \prime }\delta_{\tau ,\tau \prime }-g_{ijkl}^{\left(2\right)}\delta_{\sigma ,\tau \prime }\delta_{\tau ,\sigma \prime }){â}_{i,\sigma }^{†}{â}_{j,\tau }^{†}{â}_{l,\tau \prime }{â}_{k,\sigma \prime }$$
32

where (*i*, σ) < (*j*, τ) is understood as lexicographical ordering, and the new coefficient tensors are

$$g_{ijkl}^{\left(1\right)}=g_{ijkl}+g_{jilk}$$
33a

$$g_{ijkl}^{\left(2\right)}=g_{jikl}+g_{ijlk}$$
33b

We remark that the representation in eq 32 only uses the anticommuting property of the Fermionic operators and does not require any symmetry assumptions on the $g_{ijkl}$ tensor.

The state diagram in Figure 5 contains two terminal vertices on the left and right end, corresponding to (dummy) virtual bonds of dimension 1. The scaffolding consists of:

- Identity strings that start from the terminal vertices on either end and run to the other side.
- Single creation and annihilation operators *â*_*i*,σ_^†^ and *â*_*i*,σ_ for all *i* ∈ {0, ···, *L* – 1} and σ ∈ {↑, ↓}, starting from both sides. To facilitate connections to other operators, we extend the strings throughout the system (using local *Z* operators based on the Jordan-Wigner transform). For example, *â*_1, ↑_^†^ starting from the left is represented as
  $${â}_{1,↑}^{†}=I⊗c_{↑}^{†}Z_{↓}⊗Z_{↑}Z_{↓}⊗Z_{↑}Z_{↓}⊗...$$
  34
  where *c*_↑_^†^ is the local (bosonic) creation operator acting on the spin-up sector. Explicitly,
  $$c_{\sigma }=\left(\begin{array}{ll} 0 & 1\\ 0 & 0 \end{array}\right),,c_{\sigma }^{†}=\left(\begin{array}{ll} 0 & 0\\ 1 & 0 \end{array}\right),,\sigma \in \left\{↑,↓\right\}$$
  35
  The enumeration of occupation patterns at site l described above implies that products of local operators acting on the spin-up and spin-down sectors, like *c*_↑_^†^ *Z*_*↓*_, are Kronecker products: $c_{↑}^{†}Z_{↓}=\left(\begin{array}{cc} 0 & 0\\ 1 & 0 \end{array}\right)⊗\left(\begin{array}{cc} 1 & 0\\ 0 & -1 \end{array}\right)$ . The operator string of *â*_*i*,σ_^†^ and *â*_*i*,σ_ is connected to the identity string of its originating direction, thus avoiding a separate copy of local identity operators.
- Quadratic Fermionic operators: *â*_*i*,σ_^†^ *â*_*j*,τ_^†^ for (*i*, σ) < (*j*, τ), *â*_*j*,τ_ *â*_*i*,σ_ for (*i*, σ) < (*j*, τ), and *â*_*i*,σ_^†^ *â*_*j*,τ_. For all of these, the orbital indices *i* and *j* are both either in the left half $\left\{0,...,\left\lfloor \frac{L}{2}\right\rfloor -1\right\}$ or the right half $\left\{\left\lfloor \frac{L}{2}\right\rfloor +1,...,L-1\right\}$ , where ⌊·⌋ is the floor function. Figure 5 shows some instances of the quadratic Fermionic operator strings.

The scaffolding contains all state diagram vertices required for the MPO construction and thus also determines the virtual bond dimensions.

In the second phase, one integrates the Hamiltonian terms by adding and connecting local operators, shown in green in Figure 5. For example, the kinetic term *h*_01_ *â*_0,↑_^†^ *â*_1,↑_ is implemented by adding a state diagram edge containing the local operator *h*_01_ *c*_↑_ *I*_↓_ at site 1 and connecting this edge to the closest vertex of the string for *â*_0, ↑_^†^ on the left and to the closest vertex of the identity string attached to the right terminal on the right. Note that the Hamiltonian coefficients are solely contained in the (green) local operators added during the second phase.

During the two-site DMRG sweep for optimizing a neighboring pair of MPS tensors, the computational cost for applying the local effective Hamiltonian scales as $O\left(M^{2}D^{2}+M^{3}D\right)$ *when assuming dense tensors*, where *M* and *D* denote the maximum bond dimensions of the MPS and MPO, respectively. For a molecular Hamiltonian and *L* orbitals, $D=O\left(L^{2}\right)$ . A DMRG sweep over all orbitals incurs another complexity factor of *L*, leading to an ostensible complexity scaling of $O\left(M^{2}L^{5}+M^{3}L^{3}\right)$ . However, as noted in ref 44., the expensive $O\left(M^{2}L^{5}\right)$ computation is actually reduced to $O\left(M^{2}L^{4}\right)$ when exploiting the sparsity of the molecular MPO tensors. In our current implementation,^33^ we exploit sparsity due to particle and spin conservation, but not yet the particular sparsity pattern due to a molecular Hamiltonian. We plan to add this feature in a future update of the code, and can use the existing data structures for the above state diagram construction for this purpose.

To compute energy gradients with respect to the Hamiltonian parameters, we first use the Hellmann–Feynman theorem: assuming that the state |ψ⟩ represented as MPS is an eigenstate of the Hamiltonian *Ĥ*, then

$$\frac{\partial E}{\partial h_{ij}}=\left\langle \psi \right|\frac{\partial Ĥ}{\partial h_{ij}}\left|\psi \right\rangle$$
36a

$$\frac{\partial E}{\partial g_{ijkl}}=\sum_{i^{\prime },j^{\prime },k^{\prime },l^{\prime }}\langle \psi |\frac{\partial Ĥ}{\partial g_{i^{\prime }j^{\prime }k^{\prime }l^{\prime }}^{\left(1\right)}}|\psi \rangle \frac{\partial g_{i^{\prime }j^{\prime }k^{\prime }l^{\prime }}^{\left(1\right)}}{\partial g_{ijkl}}+\langle \psi |\frac{\partial Ĥ}{\partial g_{i^{\prime }j^{\prime }k^{\prime }l^{\prime }}^{\left(2\right)}}|\psi \rangle \frac{\partial g_{i^{\prime }j^{\prime }k^{\prime }l^{\prime }}^{\left(2\right)}}{\partial g_{ijkl}}$$
36b

We evaluate the right sides using the tensor network representation. Denoting the *l*th MPO tensor of *Ĥ* by *A* ^(*l*)^ with entries addressed by a collective index *J* (physical and virtual bond indices), then

$$\langle \psi |\frac{\partial Ĥ}{\partial h_{ij}}|\psi \rangle =\sum_{l=0}^{L-1}\sum_{J}\langle \psi |\frac{\partial Ĥ}{\partial A_{J}^{\left(l\right)}}|\psi \rangle \frac{\partial A_{J}^{\left(l\right)}}{\partial h_{ij}}$$
37

­(and analogously for the interaction coefficients). The expression $\left\langle \psi |\frac{\partial \hat{H}}{\partial A_{J}^{\left(l\right)}}|\psi \right\rangle$ (for all index values *J*) corresponds to omitting *A* ^(*l*)^ from the tensor network diagram representing ⟨ψ|*Ĥ*|ψ⟩, as schematically shown in Figure 6. This gradient tensor can be efficiently computed, analogous to the effective Hamiltonian in DMRG. The derivative $\frac{\partial A_{J}^{\left(l\right)}}{\partial h_{ij}}$ corresponds to one of the green local operators in Figure 5 without the coefficient *h*_*ij*_. We have combined these steps for computing energy gradients in chemtensor.^33^

The steps outlined here naturally follow from the chain rule and using the MPO form of the Hamiltonian. It is well-known that the energy gradients with respect to the single- and two-body coefficients are equal to the reduced density matrices, as described above in eq 11a and eq 11b. An algorithm for computing RDMs via tensor network methods has been developed in the literature before,^49^ with computational complexity $O\left(M^{3}L^{2}+M^{2}L^{4}\right)$ . Interestingly, one rediscovers their algorithm from our chain rule derivation, when additionally taking the state diagram construction in Figure 5 for the MPO tensors $A^{\left(l\right)}$ into account. In particular, $\left\langle \psi |\frac{\partial Ĥ}{\partial A_{J}^{\left(l\right)}}|\psi \right\rangle$ does not need to be evaluated as a dense tensor; instead, only the entries corresponding to the green local operators are relevant, since they depend on the Hamiltonian coefficients.

## Appendix B: MRA+DMRG Convergence Plots

In this section, we report the convergence plots for the systems simulated in Section 3. While experimenting with the algorithm, we have considered different starting basis sets:

- Standard atomic orbital basis sets, such as STO-3G or cc-pVDZ. Given the separability of the Gaussians defining the orbitals, the initialization is very fast (even if often inaccurate).
- Slater-type orbitals. More accurate than the Gaussian basis sets, they are not separable and, therefore, require more time to be initialized in an MRA framework.
- Augmented or truncated atomic orbital basis sets. This often gives a poor starting guess.
- Augmented basis sets from previously converged MRA orbitals. Good starting guess, it does not guarantee a faster convergence.

In all cases, the plots in Figure 7 show a smooth convergence to energy precision δ = 10^–5^ Ha.

While some cases like He and HeH_2_ with 14 and 6 orbitals, respectively, show a particularly poor initial guess, the optimization still manages to converge in a limited number of iterations. The N_2_ experiment with 14 orbitals is the most complicated system we have dealt with so far, given the relatively high number of configurations and the molecule’s multiconfigurational character. For this reason, we augmented the previously converged 12 orbitals basis as a starting guess.

In conclusion, we observe that the MRA+DMRG algorithm has only a weak dependence on the starting orbitals, at least for such small systems.
